# Prosthetic-Valve Endocarditis with Discordant Isolates: A Case Report and a Review of the Literature

**DOI:** 10.3390/idr18010017

**Published:** 2026-02-12

**Authors:** Raffaele Ferri, Francesco Mucedola, Marcella Conserva, Jacopo Vecchiet, Katia Falasca

**Affiliations:** Infectious Disease Unit, Azienda Sanitaria Locale Lanciano Vasto Chieti and Gabriele D’Annunzio University, 66013 Chieti, Italy; fra.mucedola@gmail.com (F.M.); conservamarcella@gmail.com (M.C.); jacopo.vecchiet@unich.it (J.V.); k.falasca@unich.it (K.F.)

**Keywords:** infective endocarditis, discordant isolates, *Streptococcus acidominimus*

## Abstract

Prosthetic-valve endocarditis (PVE) represents one of the most serious forms of infective endocarditis, marked by high mortality and considerable management complexity. The 2023 European Society of Cardiology (ESC) Guidelines emphasise the diagnostic centrality of repeatedly positive blood cultures. Nonetheless, a significant area of uncertainty remains regarding the diagnostic and prognostic value of cultures from explanted prosthetic valves—particularly in centres lacking access to molecular diagnostics. Case Presentation: We report a case of prosthetic-valve endocarditis on a bioprosthesis, in which repeated blood-culture sets yielded *Streptococcus acidominimus*, whereas culture of the explanted valve revealed *Staphylococcus warnerii*. The patient received six weeks of intravenous vancomycin, with treatment tailored according to the patient’s clinical and laboratory parameters and in alignment with international endocarditis guidelines, obtaining a clear clinical and laboratory improvement. Discussion: The literature reports that discordance between blood-culture and valve-culture results in infective endocarditis may range from approximately 10% to 29%, attributable to contamination, biofilm formation or polymicrobial infection. In our case, management guided by the microorganism repeatedly isolated from blood cultures proved effective and aligned with the 2023 European Society of Cardiology (ESC) guidelines. The case underlines the importance of a multidisciplinary team and an integrated interpretation of microbiological, clinical and surgical data. Conclusions: Infective endocarditis with discordant isolates presents a complex diagnostic challenge. The etiological diagnosis must rely primarily on the results of blood cultures, whereas valve culture plays a complementary role—useful more for prognostic stratification than for initial diagnostic purposes. A multidisciplinary approach and a critical interpretation of microbiological findings are essential to optimise therapeutic management and improve patient outcomes.

## 1. Introduction

Prosthetic-valve endocarditis (PVE) represents, according to the 2023 European Society of Cardiology (ESC) guidelines, the form of infective endocarditis associated with the poorest clinical outcomes. It occurs in approximately 1–6% of patients with prosthetic valves, with an annual incidence of 0.3–1.2% per patient-year [1]. Compared with native-valve endocarditis (NVE), PVE carries significantly higher rates of morbidity and mortality, with in-hospital mortality reported between 20% and 40%, and a persistent negative impact on long-term survival [1].

Several clinical factors have been linked to an unfavourable prognosis, including advanced age [2], diabetes mellitus, healthcare-associated acquisition, early-onset PVE, renal failure [2], and the lack of indicated surgical intervention [3]. Additional predictors of adverse outcomes include haemodynamic instability, septic shock [3], multivalvular involvement, and infection extending to the aorto-mitral fibrosa [1].

The ESC guidelines list positive blood cultures among Duke’s major diagnostic criteria and emphasise the value of molecular methods—such as Polymerase Chain Reaction (PCR) on valve tissue or embolic material—particularly in cases of culture-negative endocarditis. However, uncertainty remains regarding the true diagnostic contribution of conventional cultures of excised prosthetic valves, which retain a practical role in centres where molecular diagnostics are not yet available.

The present case aims to highlight not only a rare instance of infective endocarditis caused by *Streptococcus acidominimus* but also the diagnostic and therapeutic implications of discordant microbiological findings between blood and valve cultures—an issue that continues to challenge clinical management, particularly in institutions lacking molecular confirmation methods.

## 2. Clinical Case

### 2.1. Anamnesis and Clinical Presentation

A 37-year-old man presented to the Emergency Department with a several-week history of intermittent fever and progressive dyspnoea. His medical history was significant for prior hepatitis C virus (HCV) infection, successfully treated with direct-acting antivirals; substance use disorder in long-term remission (three years), currently managed with levomethadone maintenance therapy; and two previous episodes of infective endocarditis.

In 2023, he was diagnosed with native aortic valve endocarditis caused by *Enterococcus faecalis*. He underwent antimicrobial therapy with ampicillin and subsequently required surgical intervention with bioprosthetic aortic valve replacement. In February 2025, he experienced a new episode of prosthetic aortic valve endocarditis due to viridans group streptococci (VGS), complicated by the development of an annular abscess. On that occasion, he underwent redo cardiac surgery with implantation of a new Resilia 21 aortic bioprosthesis. Antimicrobial therapy was initially started with ceftriaxone in combination with gentamicin; however, due to an intolerance to ceftriaxone (cutaneous rash), treatment was later switched to vancomycin.

### 2.2. Diagnostic and Microbiological Procedure

On admission, laboratory tests revealed neutrophilic leukocytosis (White Blood Cells 15,310/mm^3^; neutrophils 12,270/mm^3^) and elevated inflammation markers (C-Reactive Protein 77.62 mg/L). Transthoracic echocardiography performed upon admission to the Emergency Department raised the suspicion of infective endocarditis involving the aortic bioprosthesis, revealing significant valvular dysfunction. Confirmatory transoesophageal echocardiography demonstrated a perianastomotic pseudoaneurysm or contained abscess, located between the left coronary sinus of Valsalva and the left atrial appendage, measuring approximately 21 × 10 mm in maximal axial dimensions and 22 mm longitudinally. In addition, a marked thickening of the prosthetic cusps was observed, with small vegetations protruding into the left ventricular outflow tract (LVOT), resulting in severe valvular stenosis. After the collection of culture specimens, empirical antibiotic therapy was initiated with intravenous daptomycin, gentamicin, and, after 72 h, rifampicin—in line with the 2023 ESC guidelines for prosthetic-valve endocarditis.

During hospitalisation, the patient experienced multiple episodes of ventricular fibrillation, managed with cardiopulmonary resuscitation and defibrillation, with restoration of the sinus rhythm.

### 2.3. Treatment and Clinical Course

After four days of antibiotic therapy, the patient underwent a Bentall procedure with implantation of a 25 mm mechanical prosthesis (St. Jude) and concomitant trans-aortic edge-to-edge mitral repair. The postoperative course was complicated by an ischaemic stroke and multi-organ failure characterised by acute kidney injury (serum creatinine 4.20 mg/dL, reference range 0.84 mg/dL; estimated Glomerular Filtration Rate 17 mL/min, reference range > 60 mL/min) accompanied by severe hypokalaemia (serum potassium 2.00 mmol/L, reference range 3.5–5.10 mmol/L). Laboratory testing also revealed a pronounced elevation in hepatic enzymes (AST 651 U/L, reference range: 11–34 U/L; ALT 1371 U/L, reference range < 45 U/L; GGT 119 U/L, reference range 12–64 U/L) as well as significantly increased pancreatic enzyme levels (pancreatic amylase 1169 U/L, reference range 28–100 U/L; lipase 1119 U/L, reference range < 65 U/L). In light of the progressive hepatorenal deterioration, initiation of continuous renal replacement therapy (CRRT) was required.

Microbiological re-evaluation—including two sets of blood cultures obtained upon Emergency Department admission that were positive for *Streptococcus acidominimus* (penicillin G-resistant) (Table 1) and intra-operative valve cultures positive for *Staphylococcus warnerii* (methicillin-sensitive) (Table 2)—prompted modification of therapy. The species of the blood- and valve-culture isolates were confirmed by MALDI-TOF (Matrix-Assisted Laser Desorption/Ionisation Time-of-Flight), enabling rapid and accurate species determination. Subsequent antibiotic susceptibility testing was performed using the BD Phoenix system and susceptibility was determined according to European Committee on Antimicrobial Susceptibility Testing (EUCAST) breakpoints. Molecular diagnostic analysis of the explanted tissue could not be performed owing to the temporary unavailability of the methodology at our hospital laboratory.

Empiric coverage was modified in favour of vancomycin, adjusted for renal impairment, with gentamicin re-introduction deferred. The patient received six weeks of antibiotic therapy from the date of the first negative blood cultures, achieving progressive clinical and laboratory improvement.

After three months of hospitalisation, the patient was discharged following progressive clinical and laboratory improvement, with no clinical or echocardiographic evidence of an infective endocarditis recurrence. He is currently under regular outpatient follow-up.

Given the impossibility of monitoring vancomycin blood levels via Therapeutic Drug Monitoring (TDM), continuous laboratory monitoring of inflammatory markers and hepatic-renal function was performed.

### 2.4. Clinical Case Discussion

The etiological attribution of infective endocarditis becomes particularly challenging in cases where the valve culture yields a microorganism discordant with the blood-culture isolates. In such contexts, the diagnostic value of the valve culture must be interpreted with caution: a discrepancy of up to 36% between valve and blood isolates has been reported in the literature, probably due to intraoperative contamination [4]. Consequently, the microbiological evidence of repeatedly positive blood cultures remains the most reliable criterion for establishing etiological diagnosis and guiding targeted therapy.

*Streptococcus acidominimus* is a Gram-positive coccus belonging to the viridans group streptococci (VGS). Although it is typically considered an environmental or animal commensal, human infections are exceptionally rare. A review of the literature describes limited cases of pneumonia, pericarditis, meningitis, otitis media and brain abscesses attributable to this species [5]. Moreover, sporadic instances of endocarditis caused by *S. acidominimus* have been reported [6,7,8]. Despite their very low incidence, these infections highlight that *S. acidominimus*—like other streptococci—may possess the microbiological characteristics necessary for vegetation formation on cardiac valves [9].

Given the small number of *S. acidominimus* infections described in the literature, defining specific risk factors that predispose individuals to this organism remains challenging. Even so, several published reports note that affected patients often have recent exposure to healthcare settings (such as hospitalisation, invasive procedures, or haemodialysis) or an underlying malignancy [5].

Regarding infective endocarditis, case series have linked *S. acidominimus* to congenital heart disease [5,7], particularly ventricular septal defects (VSD), or to a history of aortic valve replacement [5,6,7]. A case of *S. acidominimus* infective endocarditis occurring in a patient with an atrial myxoma has also been reported [8].

*Staphylococcus warneri* is a Gram-positive, coagulase-negative staphylococcus, part of the human skin and mucosal microbiota. Its pathogenic potential becomes significant especially in the presence of host predisposition (for example immunocompromise).

Although rare, cases of infective endocarditis on the native valve of the left heart, with microbiological evidence of *S. warneri* [10], have been documented.

The deliberate decision to target therapy towards *Streptococcus acidominimus* recovered from blood cultures proved clinically appropriate and fully aligned with the European Society of Cardiology (ESC) 2023 guideline recommendations, ultimately yielding a favourable resolution of the infectious episode.

The isolation of *S. acidominimus* from two distinct sets of blood cultures supports its likely etiological involvement; however, despite blood cultures currently being considered a major microbiological criterion in the diagnostic workup of infective endocarditis, the role of conventional culture-based analysis of explanted tissue remains controversial [11]. Indeed, the positivity of a tissue culture, particularly when the isolate differs from that obtained from blood cultures, may reflect a wide range of possibilities, from simple contamination to true polymicrobial infection.

The inability to apply molecular diagnostic techniques in the present case prevented a definitive clarification of the potential role of *Staphylococcus warneri* isolated from the excised valvular tissue, and also precluded an unequivocal determination of *S. acidominimus* as the primary pathogen. These limitations necessitated a complex etiological assessment, although both isolates exhibited antimicrobial susceptibility consistent with the therapeutic regimen administered.

The use of 16S rDNA sequencing could have represented an adjunctive tool for microbiological identification; however, genomic amplification may lead to the detection of genetic material from non-viable bacteria, potentially resulting in false-positive findings. In recent years, several studies have evaluated the integration of 16S rDNA sequencing with fluorescence in situ hybridisation (FISHseq), enabling direct identification of the causative microorganism from excised valve histological specimens and clarifying its pathogenic role. This combined approach has therefore emerged as a valuable adjunct to conventional blood cultures [12,13].

In routine clinical practice, not all institutions have the capacity to perform confirmatory molecular diagnostics when discrepancies arise between blood-culture and tissue-culture isolates or when blood cultures are negative. In such circumstances, it would be desirable to employ molecular testing selectively, particularly in cases where discordant isolates exhibit differing resistance profiles, in order to more accurately guide the choice of antimicrobial therapy.

## 3. Overview of the Literature

The available literature on infective endocarditis in which discordant microbiological isolates are found between blood cultures and valve-tissue cultures is relatively limited, although it carries important diagnostic and therapeutic implications. In routine clinical practice, valve-tissue culture has long been used as a complementary investigation in patients undergoing valve replacement, yet its diagnostic value remains controversial—particularly in cases such as the one presented, where microbial isolation from the valve differs to that from the bloodstream. Several studies have demonstrated that this discordance, reported in varying proportions, may be due to intraoperative contamination, polymicrobial infections, or colonisation without pathogenic significance. However, in more complex clinical settings, the interpretation of such results can be challenging and may significantly influence the therapeutic strategy.

In recent years, the introduction of molecular diagnostics such as PCR and 16S-rRNA sequencing has enhanced the etiological investigation of infective endocarditis (IE)—particularly in cases of culture-negative blood results or when isolates from valve tissue and blood are discordant. However, the availability of these methods remains limited in many centres, and their results must be interpreted within the full clinical–microbiological context. In light of these developments, it is useful to critically review the principal contributions in the literature concerning discordance between blood- and valve-culture isolates in IE—focusing on their diagnostic significance, therapeutic implications and alignment with major international guideline recommendations.

We conducted an overview of the literature from the past 20 years focusing on adult patients, in whom microbiological results from explanted valve tissue were compared with those from blood cultures.

As early as 2008, in the study by Muñoz et al., the authors sought to define the diagnostic value of heart-valve culture in patients both with and without infective endocarditis and emphasised that, in confirmed cases of endocarditis, the result of valve culture must be interpreted with extreme caution [11]. In that series, 71 excised valves from patients with endocarditis were studied. Among these, there were 10 cases in which a discrepancy between blood-culture and valve-culture results was documented. The authors attributed such discrepancies less to intrinsic methodological errors and more to potential contamination during surgery, sample-handling issues, or laboratory processing phases, emphasising that while valve culture may provide supportive diagnostic information in infective endocarditis (IE), its results—particularly when discordant with blood culture findings—cannot be considered conclusive for etiologic attribution when interpreted in isolation.

In keeping with the observations of Muñoz et al., Pablo Elpidio García Granja et al. [14] conducted an analysis of patients with left-sided infective endocarditis who had positive blood cultures and underwent surgery during the active phase of infection, with a microbiological assessment of surgically obtained valve tissue (*n* = 371). Among these, groups were stratified according to valve-culture results: negative valve culture (*n* = 218) and concordant positive valve culture (CPVC) (*n* = 118), with the remaining 35 patients showing discordant isolations. Despite the primary aim being prognostic stratification rather than diagnostic attribution, the authors clearly emphasised that valve-culture results offered limited diagnostic contribution (Table 1).

In the study by Johansson et al. [15], aimed at identifying the risk factors for positive valve cultures and their relationship with the outcome, 345 valves of surgical IE were analysed; valve cultures were positive in 78 cases (23%) and negative in 267 cases (77%). The authors reported eight valve cultures (10.3% of 78 positive cases) classified as contaminated. In these discordant cases, 16S rDNA analysis was performed and confirmed the blood culture-identified pathogen in seven of the eight valve-tissue samples; the remaining case was not confirmed because the analysis was not performed (Table 3).

Overall, the literature suggests that culture of the explanted valve hold only a marginal role in the diagnostic workup of infective endocarditis, with greater reliability attributed to blood cultures or—when available—molecular diagnostics, which have demonstrated a particular usefulness in cases of culture-negative endocarditis.

## 4. Conclusions

Infective endocarditis is a complex clinical condition whose diagnosis and management requires an integrated, multidisciplinary approach, particularly as discordant isolates between blood cultures and valve cultures—though not rare—pose significant interpretative challenges with important therapeutic implications.

The critical analysis of our case, supported by the literature, suggests that repeatedly positive and clinically consistent blood cultures should remain the primary reference for etiological attribution, whereas valve-culture results must be interpreted with caution—particularly when unsupported by compatible clinical evidence. In such circumstances, therapeutic decisions should be based on a comprehensive assessment that integrates laboratory, microbiological, clinical and surgical data, avoiding empiric extension of antimicrobial therapy in the absence of clear signs of active infection.

Nevertheless, valve cultures retain a complementary role, particularly with the advent and growing dissemination of molecular techniques (such as 16S-rRNA sequencing and metagenomic analysis), which hold promise for enhancing diagnostic sensitivity and clarifying the clinical significance of discordant isolates.

In settings where molecular diagnostic techniques are not readily available, clinicians should be encouraged to seek support from regional reference microbiology laboratories, which may also facilitate access to advanced diagnostics for smaller centres with limited financial resources. The referral of excised valvular tissue for molecular analysis may provide critical diagnostic and therapeutic information in selected cases, particularly in patients with blood culture-negative infective endocarditis. Such collaborative diagnostic strategies could help clarify etiologic uncertainty, optimise antimicrobial management, and ultimately improve patient outcomes.

In conclusion, the management of infective endocarditis mandates a careful integration of microbiological data within the clinical context. Our case supports the view that an approach based on a comprehensive, multidisciplinary assessment remains the cornerstone for optimising diagnosis, guiding antibiotic therapy appropriately, and improving outcomes in patients afflicted by this serious infectious complication.

## Figures and Tables

**Table 1:** Antibiotic susceptibility test with Minimal Inhibitory Concentration (MIC)—*S. acidominimus*.

Antibiotic	Interpretation	MIC
Vancomycin	S (Susceptible)	≤0.5 mg/L
Teicoplanin	S (Susceptible)	≤1 mg/L
Penicillin G	R (Resistant)	2.000
Meropenem	S (Susceptible)	2.000
Gentamicin	R (Resistant)	
Cefepime	S (Susceptible)	≤0.5 mg/L
Cefuroxime	S (Susceptible)	1
Cefotaxime	S (Susceptible)	≤0.5 mg/L
Clindamycin	S (Susceptible)	≤0.03125 mg/L
Amoxicillin	R (Resistant)	≥4.000

**Table 2:** Antibiotic susceptibility test with Minimal Inhibitory Concentration (MIC)—*S. warnerii*.

Antibiotic	Interpretation	MIC
Vancomycin	S (Susceptible)	1
Teicoplanin	S (Susceptible)	≤0.5 mg/L
Tetracycline	S (Susceptible)	≤0.5 mg/L
Trimethoprim/Sulfamethoxazole	S (Susceptible)	≤1/19 mg/L
Oxacillin	S (Susceptible)	≤0.25 mg/L
Linezolid	S (Susceptible)	1
Gentamicin	S (Susceptible)	≤1
Daptomycin	S (Susceptible)	1
Ciprofloxacin	S (Susceptible)	≤0.5 mg/L
Clindamycin	S (Susceptible)	≤0.25 mg/L
Erythromycin	R (Resistant)	≥2000
Rifampicina		≤0.25

**Table 3:** Main studies on the discordances between blood cultures and valve cultures.

Studies	Number of Patients with Positive Blood and Valve Cultures	Number of Discordant Cultures Between Blood and Valve Cultures	Percentage of Discrepancy
Munoz et al., 2008 [11]	71	10	14.1%
Granja et al., 2019 [14]	118	35	29.7%
Johansson et al., 2024 [15]	78	8	10.3%

## Data Availability

The original contributions presented in this study are included in the article. Further inquiries can be directed to the corresponding author.

## Abbreviations

The following abbreviations are used in this manuscript:

PVE	Prosthetic-valve endocarditis
ESC	European Society of Cardiology
NVE	Native-valve endocarditis
PCR	Polymerase Chain Reaction
CRP	Reactive C-Protein
MIC	Minimal Inhibitory Concentration
FISH	fluorescence in situ hybridisation
VGS	Viridans Group Streptococci
